# The genome sequence of an ichneumonid wasp, *Exephanes ischioxanthus *(Gravenhorst, 1829)

**DOI:** 10.12688/wellcomeopenres.20498.1

**Published:** 2024-01-08

**Authors:** Gavin R. Broad, Natural History Museum Genome Acquisition Lab, Darwin Tree of Life Barcoding collective, Wellcome Sanger Institute Tree of Life Management, Samples and Laboratory team, Wellcome Sanger Institute Scientific Operations: Sequencing Operations, Wellcome Sanger Institute Tree of Life Core Informatics team, Tree of Life Core Informatics collective, Darwin Tree of Life Consortium

**Affiliations:** 1 Natural History Museum, London, England, UK

**Keywords:** Exephanes ischioxanthus, an ichneumonid wasp, genome sequence, chromosomal, Hymenoptera

## Abstract

We present a genome assembly from an individual male *Exephanes ischioxanthus* (an ichneumonid wasp; Arthropoda; Insecta; Hymenoptera; Ichneumonidae). The genome sequence is 284.0 megabases in span. Most of the assembly is scaffolded into 12 chromosomal pseudomolecules. The mitochondrial genome has also been assembled and is 19.43 kilobases in length.

## Species taxonomy

Eukaryota; Metazoa; Eumetazoa; Bilateria; Protostomia; Ecdysozoa; Panarthropoda; Arthropoda; Mandibulata; Pancrustacea; Hexapoda; Insecta; Dicondylia; Pterygota; Neoptera; Endopterygota; Hymenoptera; Apocrita; Ichneumonoidea; Ichneumonidae; Ichneumoninae; Ichneumonini; *Exephanes*; *Exephanes ischioxanthus* (Gravenhorst, 1829) (NCBI:txid2884240).

## Background

*Exephanes ischioxanthus* is an ichneumonid, or ‘Darwin wasp’, widely distributed and frequently common in grassy areas. It is found across much of Europe ( Yu *et al.*, 2016), with adults on the wing in summer, particularly June and July. As with many species of the subfamily Ichneumoninae, females are mainly black but with the metasoma (abdomen beyond the first segment) red medially and with white spots on the antennae, scutellum and the posterior of the metasoma. However, the ovipositor projects further than in most Ichneumoninae and the end of the metasoma is a little elongated, with eight metasomal tergites clearly visible. Males look very different, with the metasoma yellow centrally and the face entirely yellow. Specimens can be easily identified using Perkins (1960), but Hinz and Horstmann (2000) offer a more reliable identification key to the European *Exephanes*, with information on ecology.

The distinctive metasoma and ovipositor of *Exephanes* species are adaptations towards oviposition into larvae of noctuid moth larvae in grass stems and *E. ischioxanthus* is a parasitoid of *Mesoligia furuncula* (Denis & Schiffermüller), the Cloaked Minor ( Hinz & Horstmann, 2000). Oviposition is into the larva and emergence, as in almost all Ichneumoninae, is from the host pupa ( Broad *et al.*, 2018). Both sexes can be found feeding on flowers and the sequenced individual was a male found on an umbel. Females have been found swarming over low vegetation in warm, humid weather just before storms (G. Broad, pers. obs.).

## Genome sequence report

**Figure 1.**
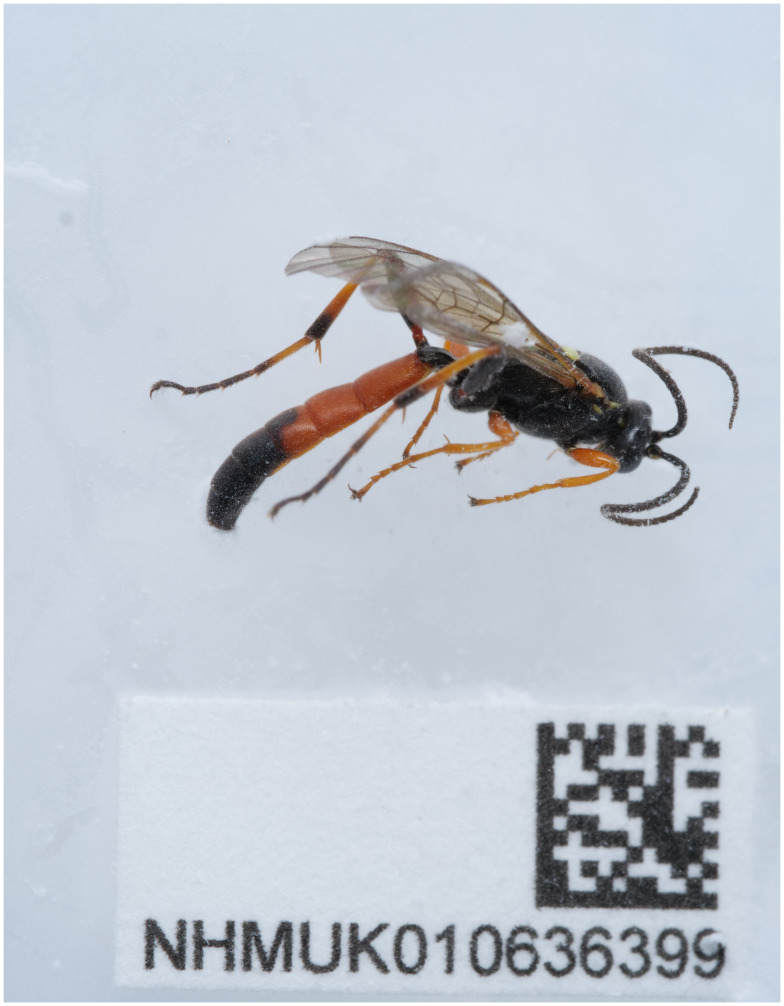
Photograph of the *Exephanes ischioxanthus* (iyExeIsch1) specimen used for genome sequencing.

The genome was sequenced from one male *Exephanes ischioxanthus* ( Figure 1) collected from Oare Marshes, England, UK (51.34, 0.89). A total of 30-fold coverage in Pacific Biosciences single-molecule HiFi long reads was generated. Primary assembly contigs were scaffolded with chromosome conformation Hi-C data. Manual assembly curation corrected 110 missing joins or mis-joins, reducing the scaffold number by 37.5%, and increasing the scaffold N50 by 22.81%.

**Figure 2.**
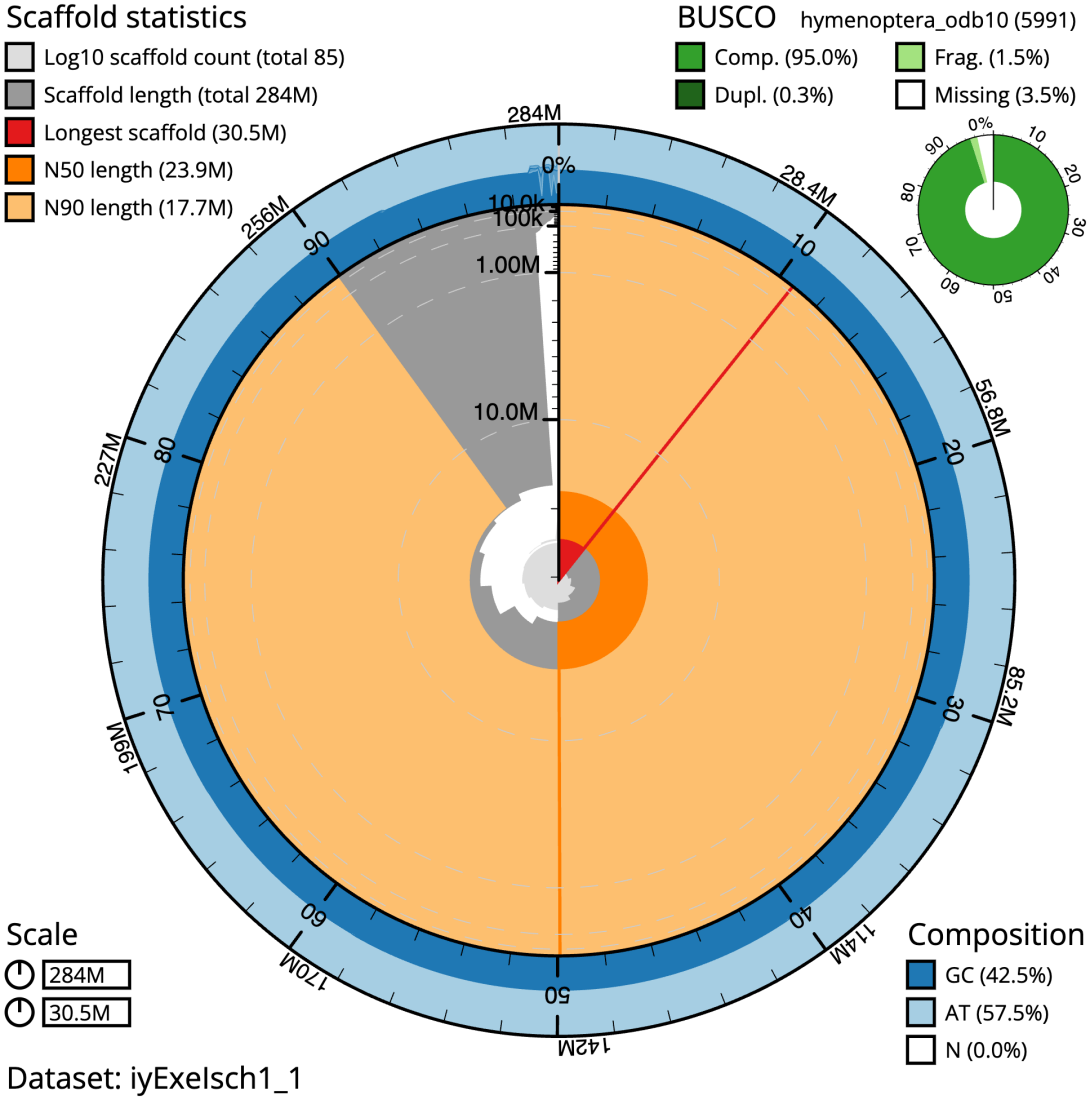
Genome assembly of *Exephanes ischioxanthus*, iyExeIsch1.1: metrics. The BlobToolKit Snailplot shows N50 metrics and BUSCO gene completeness. The main plot is divided into 1,000 size-ordered bins around the circumference with each bin representing 0.1% of the 284,017,360 bp assembly. The distribution of scaffold lengths is shown in dark grey with the plot radius scaled to the longest scaffold present in the assembly (30,499,120 bp, shown in red). Orange and pale-orange arcs show the N50 and N90 scaffold lengths (23,927,040 and 17,743,748 bp), respectively. The pale grey spiral shows the cumulative scaffold count on a log scale with white scale lines showing successive orders of magnitude. The blue and pale-blue area around the outside of the plot shows the distribution of GC, AT and N percentages in the same bins as the inner plot. A summary of complete, fragmented, duplicated and missing BUSCO genes in the hymenoptera_odb10 set is shown in the top right. An interactive version of this figure is available at https://blobtoolkit.genomehubs.org/view/Exephanes%20ischioxanthus/dataset/iyExeIsch1_1/snail.

**Figure 3.**
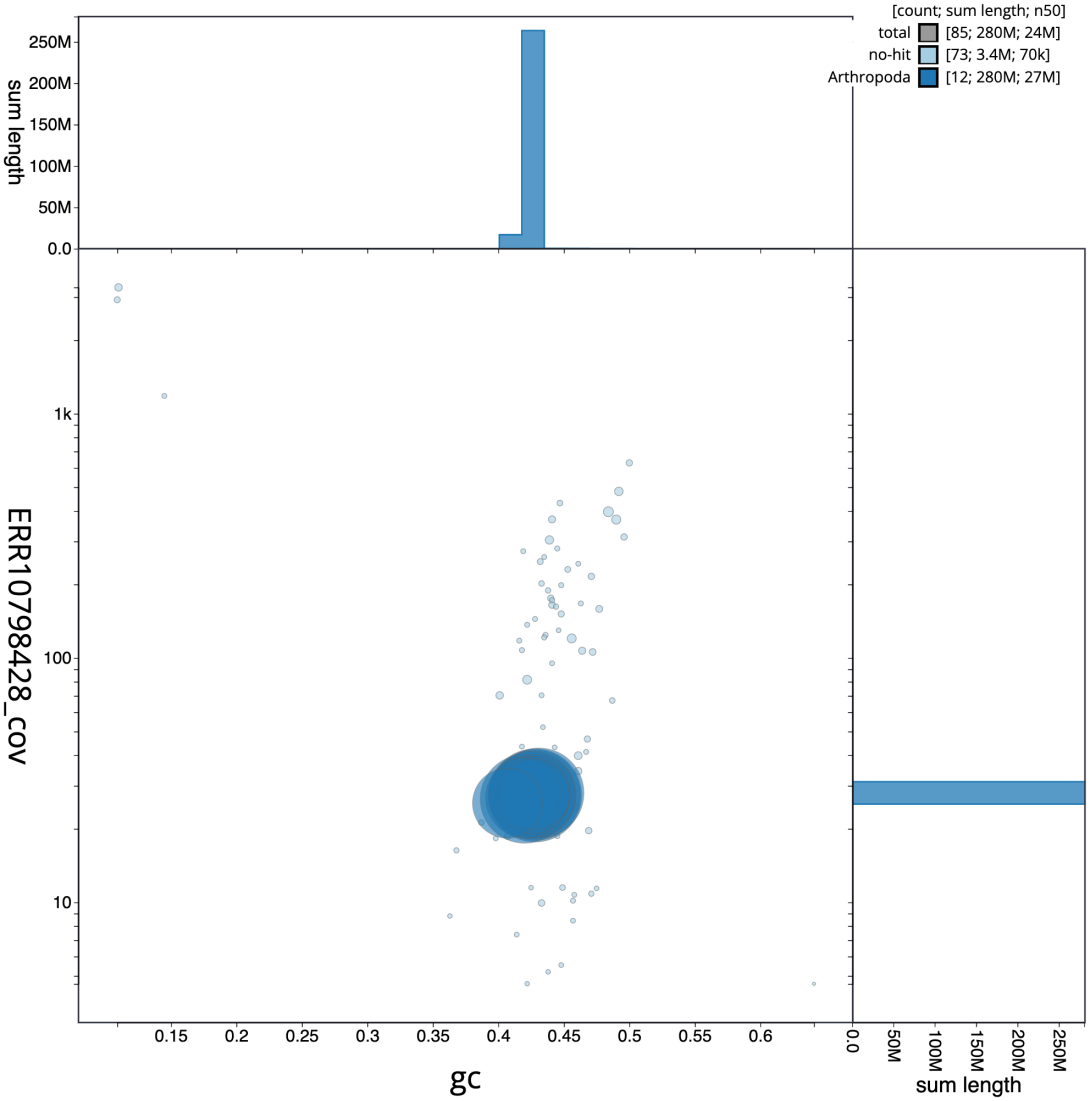
Genome assembly of *Exephanes ischioxanthus*, iyExeIsch1.1: BlobToolKit GC-coverage plot. Scaffolds are coloured by phylum. Circles are sized in proportion to scaffold length. Histograms show the distribution of scaffold length sum along each axis. An interactive version of this figure is available at https://blobtoolkit.genomehubs.org/view/Exephanes%20ischioxanthus/dataset/iyExeIsch1_1/blob.

**Figure 4.**
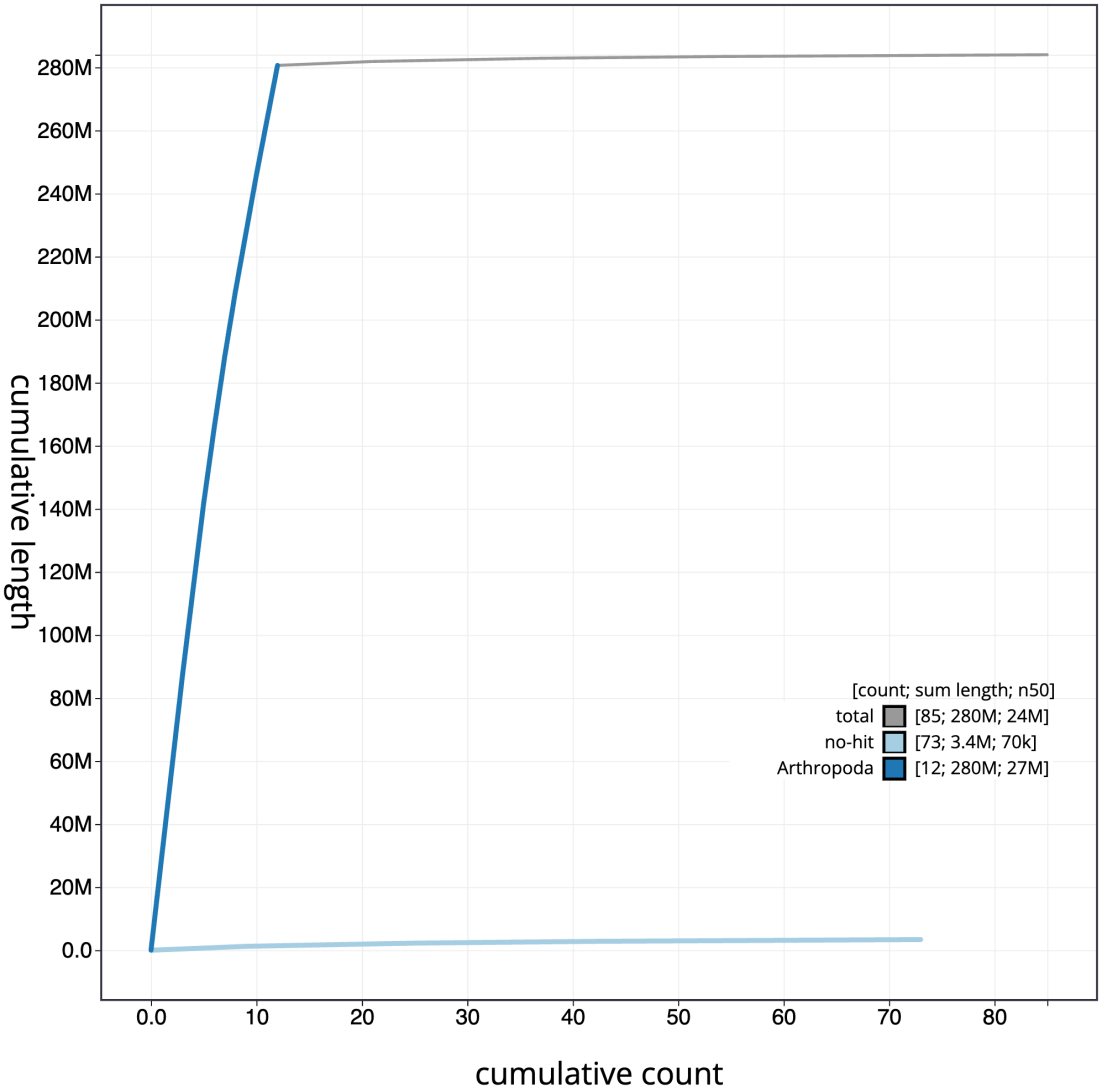
Genome assembly of *Exephanes ischioxanthus*, iyExeIsch1.1: BlobToolKit cumulative sequence plot. The grey line shows cumulative length for all scaffolds. Coloured lines show cumulative lengths of scaffolds assigned to each phylum using the buscogenes taxrule. An interactive version of this figure is available at https://blobtoolkit.genomehubs.org/view/Exephanes%20ischioxanthus/dataset/iyExeIsch1_1/cumulative.

**Figure 5.**
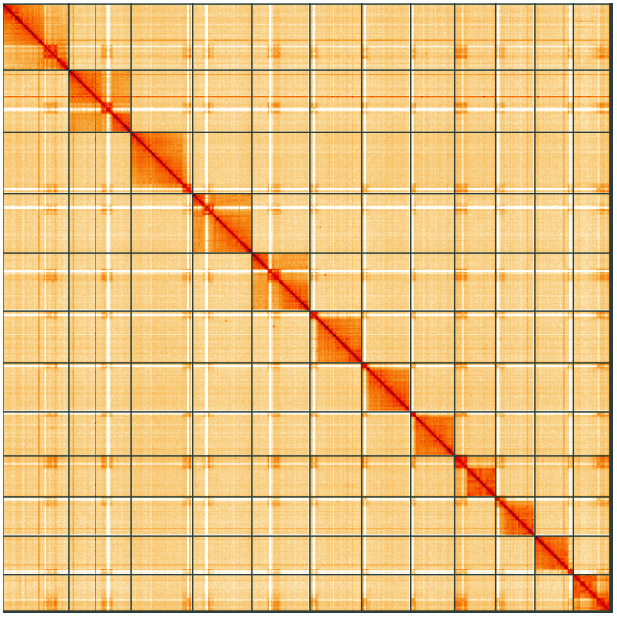
Genome assembly of *Exephanes ischioxanthus*, iyExeIsch1.1: Hi-C contact map of the iyExeIsch1.1 assembly, visualised using HiGlass. Chromosomes are shown in order of size from left to right and top to bottom. An interactive version of this figure may be viewed at https://genome-note-higlass.tol.sanger.ac.uk/l/?d=GVHDG0BnSbWn8WvXMQdKVw.

The final assembly has a total length of 284.0 Mb in 84 sequence scaffolds with a scaffold N50 of 23.9 Mb ( Table 1). The snailplot in Figure 2 provides a summary of the assembly statistics, while the distribution of assembly scaffolds on GC proportion and coverage is shown in Figure 3. The cumulative assembly plot in Figure 4 shows curves for subsets of scaffolds assigned to different phyla. Most (98.8%) of the assembly sequence was assigned to 12 chromosomal-level scaffolds. Chromosome-scale scaffolds confirmed by the Hi-C data are named in order of size ( Figure 5; Table 2). The specimen is a haploid male. The mitochondrial genome was also assembled and can be found as a contig within the multifasta file of the genome submission.

**Table 1.** Genome data for *Exephanes ischioxanthus*, iyExeIsch1.1.

Project accession data		
Assembly identifier	iyExeIsch1.1	
Species	*Exephanes ischioxanthus*	
Specimen	iyExeIsch1	
NCBI taxonomy ID	2884240	
BioProject	PRJEB58956	
BioSample ID	SAMEA110019319	
Isolate information	iyExeIsch1, male: head and thorax (DNA sequencing and Hi-C data)	
Assembly metrics *		*Benchmark*
Consensus quality (QV)	59.4	*≥ 50*
*k*-mer completeness	100%	*≥ 95%*
BUSCO **	C:95.0%[S:94.7%,D:0.3%],F:1.5%,M: 3.5%,n:5,991	*C ≥ 95%*
Percentage of assembly mapped to chromosomes	98.8%	*≥ 95%*
Sex chromosomes	-	*localised homologous pairs*
Organelles	Mitochondrial genome assembled	*complete single alleles*
Raw data accessions		
PacificBiosciences SEQUEL II	ERR10798428	
Hi-C Illumina	ERR10786029	
Genome assembly		
Assembly accession	GCA_958510785.1	
Span (Mb)	284.0	
Number of contigs	544	
Contig N50 length (Mb)	1.1	
Number of scaffolds	84	
Scaffold N50 length (Mb)	23.9	
Longest scaffold (Mb)	30.5	

* Assembly metric benchmarks are adapted from column VGP-2020 of “Table 1: Proposed standards and metrics for defining genome assembly quality” from ( Rhie *et al.*, 2021). ** BUSCO scores based on the hymenoptera_odb10 BUSCO set using v5.3.2. C = complete [S = single copy, D = duplicated], F = fragmented, M = missing, n = number of orthologues in comparison. A full set of BUSCO scores is available at https://blobtoolkit.genomehubs.org/view/Exephanes%20ischioxanthus/dataset/iyExeIsch1_1/busco.

**Table 2.** Chromosomal pseudomolecules in the genome assembly of *Exephanes ischioxanthus*, iyExeIsch1.

INSDC accession	Chromosome	Length (Mb)	GC%
OY294015.1	1	30.5	43.0
OY294016.1	2	28.8	43.0
OY294017.1	3	28.35	43.0
OY294018.1	4	27.48	42.0
OY294019.1	5	26.8	42.5
OY294020.1	6	23.93	42.5
OY294021.1	7	22.62	42.0
OY294022.1	8	20.37	42.5
OY294023.1	9	18.95	42.5
OY294024.1	10	18.21	42.0
OY294025.1	11	17.74	42.5
OY294026.1	12	16.9	40.5
OY294027.1	MT	0.02	14.5

The estimated Quality Value (QV) of the final assembly is 59.4 with *k*-mer completeness of 100%, and the assembly has a BUSCO v5.3.2 completeness of 95.0% (single = 94.7%, duplicated = 0.3%), using the hymenoptera_odb10 reference set ( *n* = 5,991).

Metadata for specimens, barcode results, spectra estimates, sequencing runs, contaminants and pre-curation assembly statistics are given at https://links.tol.sanger.ac.uk/species/2884240.

## Methods

### Sample acquisition and nucleic acid extraction

A male *Exephanes ischioxanthus* (specimen ID NHMUK010636399, ToLID iyExeIsch1) was collected from Oare Marshes, England UK (latitude 51.34, longitude 0.89) on 2021-06-20 using an aerial net. The specimen was collected and identified by Gavin Broad (Natural History Museum) and preserved by dry freezing at –80 °C.

The workflow for high molecular weight (HMW) DNA extraction at the Wellcome Sanger Institute (WSI) includes a sequence of core procedures: sample preparation; sample homogenisation; DNA extraction; HMW DNA fragmentation; and fragmented DNA clean-up. The sample was prepared for DNA extraction at the WSI Tree of Life laboratory: the iyExeIsch1 sample was weighed and dissected on dry ice with tissue set aside for Hi-C sequencing ( https://dx.doi.org/10.17504/protocols.io.x54v9prmqg3e/v1). Tissue from the head and organism was disrupted using a Nippi Powermasher fitted with a BioMasher pestle ( https://dx.doi.org/10.17504/protocols.io.5qpvo3r19v4o/v1). DNA was extracted at the WSI Scientific Operations core using the Qiagen MagAttract HMW DNA kit, according to the manufacturer’s instructions.

Protocols developed in the Tree of Life laboratory are publicly available on protocols.io: https://dx.doi.org/10.17504/protocols.io.8epv5xxy6g1b/v1.

### Sequencing

Pacific Biosciences HiFi circular consensus DNA sequencing libraries were constructed according to the manufacturers’ instructions. DNA sequencing was performed by the Scientific Operations core at the WSI on a Pacific Biosciences SEQUEL II instrument. Hi-C data were also generated from remaining head and thorax tissue of iyExeIsch1 using the Arima2 kit and sequenced on the Illumina NovaSeq 6000 instrument.

### Genome assembly, curation and evaluation

Assembly was carried out with Hifiasm ( Cheng *et al.*, 2021). The assembly was then scaffolded with Hi-C data ( Rao *et al.*, 2014) using YaHS ( Zhou *et al.*, 2023). The assembly was checked for contamination and corrected as described previously ( Howe *et al.*, 2021). Manual curation was performed using HiGlass ( Kerpedjiev *et al.*, 2018) and Pretext ( Harry, 2022). The mitochondrial genome was assembled using MitoHiFi ( Uliano-Silva *et al.*, 2023), which runs MitoFinder ( Allio *et al.*, 2020) or MITOS ( Bernt *et al.*, 2013) and uses these annotations to select the final mitochondrial contig and to ensure the general quality of the sequence.

A Hi-C map for the final assembly was produced using bwa-mem2 ( Vasimuddin *et al.*, 2019) in the Cooler file format ( Abdennur & Mirny, 2020). To assess the assembly metrics, the *k*-mer completeness and QV consensus quality values were calculated in Merqury ( Rhie *et al.*, 2020). This work was done using Nextflow ( Di Tommaso *et al.*, 2017) DSL2 pipelines “sanger-tol/readmapping” ( Surana *et al.*, 2023a) and “sanger-tol/genomenote” ( Surana *et al.*, 2023b). The genome was analysed within the BlobToolKit environment ( Challis *et al.*, 2020) and BUSCO scores ( Manni *et al.*, 2021; Simão *et al.*, 2015) were calculated.

Table 3 contains a list of relevant software tool versions and sources.

**Table 3.** Software tools: versions and sources.

Software tool	Version	Source
BlobToolKit	4.1.7	https://github.com/blobtoolkit/blobtoolkit
BUSCO	5.3.2	https://gitlab.com/ezlab/busco
Hifiasm	0.16.1-r375	https://github.com/chhylp123/hifiasm
HiGlass	1.11.6	https://github.com/higlass/higlass
Merqury	MerquryFK	https://github.com/thegenemyers/MERQURY.FK
MitoHiFi	2	https://github.com/marcelauliano/MitoHiFi
PretextView	0.2	https://github.com/wtsi-hpag/PretextView
sanger-tol/genomenote	v1.0	https://github.com/sanger-tol/genomenote
sanger-tol/ readmapping	1.1.0	https://github.com/sanger-tol/readmapping/tree/1.1.0
YaHS	1.2a	https://github.com/c-zhou/yahs

### Wellcome Sanger Institute – Legal and Governance

The materials that have contributed to this genome note have been supplied by a Darwin Tree of Life Partner. The submission of materials by a Darwin Tree of Life Partner is subject to the **‘Darwin Tree of Life Project Sampling Code of Practice’**, which can be found in full on the Darwin Tree of Life website here. By agreeing with and signing up to the Sampling Code of Practice, the Darwin Tree of Life Partner agrees they will meet the legal and ethical requirements and standards set out within this document in respect of all samples acquired for, and supplied to, the Darwin Tree of Life Project.

Further, the Wellcome Sanger Institute employs a process whereby due diligence is carried out proportionate to the nature of the materials themselves, and the circumstances under which they have been/are to be collected and provided for use. The purpose of this is to address and mitigate any potential legal and/or ethical implications of receipt and use of the materials as part of the research project, and to ensure that in doing so we align with best practice wherever possible. The overarching areas of consideration are:

•   Ethical review of provenance and sourcing of the material

•   Legality of collection, transfer and use (national and international)

Each transfer of samples is further undertaken according to a Research Collaboration Agreement or Material Transfer Agreement entered into by the Darwin Tree of Life Partner, Genome Research Limited (operating as the Wellcome Sanger Institute), and in some circumstances other Darwin Tree of Life collaborators.

## Data Availability

European Nucleotide Archive: *Exephanes ischioxanthus*. Accession number PRJEB58956; https://identifiers.org/ena.embl/PRJEB58956 ( Wellcome Sanger Institute, 2023). The genome sequence is released openly for reuse. The *Exephanes ischioxanthus* genome sequencing initiative is part of the Darwin Tree of Life (DToL) project. All raw sequence data and the assembly have been deposited in INSDC databases. The genome will be annotated using available RNA-Seq data and presented through the Ensembl pipeline at the European Bioinformatics Institute. Raw data and assembly accession identifiers are reported in Table 1.

## Author information

Members of the Natural History Museum Genome Acquisition Lab are listed here: https://doi.org/10.5281/zenodo.7139035.

Members of the Darwin Tree of Life Barcoding collective are listed here: https://doi.org/10.5281/zenodo.4893703.

Members of the Wellcome Sanger Institute Tree of Life Management, Samples and Laboratory team are listed here: https://doi.org/10.5281/zenodo.10066175.

Members of Wellcome Sanger Institute Scientific Operations: Sequencing Operations are listed here: https://doi.org/10.5281/zenodo.10043364.

Members of the Wellcome Sanger Institute Tree of Life Core Informatics team are listed here: https://doi.org/10.5281/zenodo.10066637.

Members of the Tree of Life Core Informatics collective are listed here: https://doi.org/10.5281/zenodo.5013541.

Members of the Darwin Tree of Life Consortium are listed here: https://doi.org/10.5281/zenodo.4783558.
