# Supplementary material for: The genome sequence of an ichneumonid wasp, Exephanes ischioxanthus (Gravenhorst, 1829)
Source: Wellcome Open Res. 2024 Jan 8;9:8. [Version 1] doi: 10.12688/wellcomeopenres.20498.1 (PMC11372355; doi:10.12688/wellcomeopenres.20498.1)
Supplement: Peer review history

# Reviewer report: Reviewer response for version 1

**DOI:** 10.21956/wellcomeopenres.22691.r91542

**Published:** 2024-09-03

**Authors:** Rahia Mashoodh

**Affiliations:** 1 University College London, London, England, UK

This is a detailed genome for the parasitic wasp *Exephanes ischioxanthus.* Given the sheer number of wasp species in this family, it would be nice to have a sentence or two explaining the broader interest of the species to the community. For example, for understanding genome dynamics, evolution, or ecological differences between different communities or species of this parasitic wasp.

Are sufficient details of methods and materials provided to allow replication by others?

Yes

Is the rationale for creating the dataset(s) clearly described?

Yes

Are the datasets clearly presented in a useable and accessible format?

Yes

Are the protocols appropriate and is the work technically sound?

Yes

Reviewer Expertise:

behaviour, genomics, evolution, sociality

I confirm that I have read this submission and believe that I have an appropriate level of expertise to confirm that it is of an acceptable scientific standard.

# Reviewer report: Reviewer response for version 1

**DOI:** 10.21956/wellcomeopenres.22691.r74030

**Published:** 2024-08-28

**Authors:** Sarah Inwood

**Affiliations:** 1 University of Otago, Dunedin, Otago, New Zealand

The authors sequenced and assembled a chromosomal scale genome assembly for the parasitic wasp genome of *Exephanes ischioxanthus* (Gravenhorst, 1829) using HiFi and Hi-C data. This has generated a high-quality genome assembly, using technically sound assembly and QC methods, and is well-written.

**Minor comments:**

- The significance of having a genome available for *E. ischioxanthus* is not made immediately clear to readers.
- The methods describing what tissues were used for each sequencing method (HiFi and Hi-C) could be clearer, with the head reported to be used by both methods.
- The method for mitochondrial genome assembly could also be clarified, as the authors mention using MitoHiFi which can use MitoFinder or MITOS but do not report which one was used for their assembly.

Are sufficient details of methods and materials provided to allow replication by others?

Partly

Is the rationale for creating the dataset(s) clearly described?

Yes

Are the datasets clearly presented in a useable and accessible format?

Yes

Are the protocols appropriate and is the work technically sound?

Yes

Reviewer Expertise:

Genomics, host-parasite interactions, parasitoid wasps, insects, viruses, biocontrol

I confirm that I have read this submission and believe that I have an appropriate level of expertise to confirm that it is of an acceptable scientific standard, however I have significant reservations, as outlined above.

# Reviewer report: Reviewer response for version 1

**DOI:** 10.21956/wellcomeopenres.22691.r74032

**Published:** 2024-02-27

**Authors:** Brendan Pinto

**Affiliations:** 1 Arizona State University, Tempe, AZ, USA

The authors describe the high-quality genome assembly of the Ichneumon wasp, *Exephanes ischioxanthus*. The manuscript is technically sound and uses assembly and QC pipelines that have been previously vetted through use (and reuse) by the Sanger Institute. Reference genomes are certainly important to have for investigating myriad interesting scientific questions, however, the importance of this specific genome is not made entirely clear (at least to me) for a broader readership. Regardless, the note is well-written, describes an important resource for genomics in an interesting clade of hymenopterans.

Are sufficient details of methods and materials provided to allow replication by others?

Yes

Is the rationale for creating the dataset(s) clearly described?

Partly

Are the datasets clearly presented in a useable and accessible format?

Yes

Are the protocols appropriate and is the work technically sound?

Yes

Reviewer Expertise:

My expertise is in genomics of squamate reptiles. I was also involved in the group who published one of the first chromosome-level genomes for a braconid wasp (genus Cotesia).

I confirm that I have read this submission and believe that I have an appropriate level of expertise to confirm that it is of an acceptable scientific standard.
